# Comparison of Colonic J-pouch and Straight Coloanal anastomosis after Low Anterior Resection

**DOI:** 10.5812/ircmj.3804

**Published:** 2013-01-05

**Authors:** Shaban Mehrvarz, Seyed Mohsen Towliat, Hassan Ali Mohebbi, Saieed Derakhshani, Mahdi Abavisani

**Affiliations:** 1 Department of General Surgery, Baqiyatallah University of Medical Sciences, Tehran, IR Iran; 2 Baqiyatallah University of Medical Sciences, Research Center for Gastroenterology and Liver Disease, Tehran, IR Iran; 3 Department of General Surgery, Chamran Hospital, Tehran, IR Iran

**Keywords:** Rectal Cancer, Straight Coloanal Anastomosis, Colonic J-Pouch Anal Anastomosis, Low Anterior Resection, Quality of Life

## Abstract

**Background:**

The tendency towards sphincter preserving for low rectal cancers with low anterior resection, has led to the technique of straight coloanal anastomosis (SCAA) or colonic J-pouch anal anastomosis (CPAA).

**Objectives:**

The aim of our study was to compare functional outcomes, complication rates and quality of life (QoL) after LAR with either a straight or colonic J pouch anastomosis.

**Patients and Methods:**

In 88 patients with rectal tumors located in lower third, who were candidate for LAR with coloanal anastomosis. They were divided for reconstruction using either SCAA (n= 47) or CPAA (n= 41) from January 2007 to May 2009. Functional results were assessed after closure of temporary loop ileostomy, 6 months postoperatively. Quality of life (QoL) was measured using European Organization for Research and Treatment of Cancer (EORTC) QLQ-C30.

**Results:**

The two groups were matched for gender, age, and preoperative chemotherapy and radiotherapy. There were no significant differences between the SCAA and CPAA groups relative to anastomotic leakage. Among patients with CPAA, the mean of 24 hours bowel movements, daytime bowel movements, incontinence scores, and incidence of urgency were significantly lower than those in the SCAA group. Also, patients with a CPAA had a significantly better quality of life.

**Conclusions:**

CPAA provided not only better functional results than SCAA, but also improved quality of life, thus may be the better choice.

## 1. Background

Over the last two decades, major evolutions have occurred in the treatment of rectal cancer. Currently, with advances in rectal anastomosis techniques, sphincter-saving procedure have become the standard treatment in the surgical approach to most cancers of the upper, middle, and even lower third of the rectum (1, 2) Since its description by Parks, low anterior resection (LAR) with straight coloanal anastomosis has gained wide acceptance in the treatment of the cancer of the lower third of the rectum (3, 4). Unfortunately, straight coloanal anastomosis often leads to the Anterior Resection Syndrome, characterized by increased stool frequency, urgency, and incontinence primarily due to loss of the reservoir function that was previously provided by the rectum, which lowers the quality of life (QOL) in patients undergoing resection of the distal rectum (5-9). To minimize these symptoms, Lazorthes and Parc independently described the construction of a colonic J-pouch from the distal colon (10, 11). Although a number of studies that compared the J-pouch with straight end-to-end coloanal anastomosis have shown functional superiority of the pouch, especially in the first year after surgery (10, 12-15), but still it remains uncertain which of these reconstructive techniques is superior and controversies still exist (5,16). Moreover, better functional results are not necessarily associated with better QOL, where this has been reported repeatedly in patients with various anorectal problems (2, 17, 18). Thus this study was designed to analyze the functional results and QOL of patients undergoing these two salvage techniques.

## 2. Objectives

The aim of our study was to compare functional outcomes, complication rates, QOL after LAR with either a straight or colonic J-pouch anastomosis.

## 3. Patients and Methods

These clinical trials have been achieved from January 2007 to May 2009 in Baqiyatallah and Chamran hospitals, Tehran-Iran, where a total of 88 patients with tumors located in lower rectum were included. They were candidate for LAR with coloanal anastomosis, thus they were divided to either straight Coloanal anastomosis (SCAA) or colonic J-pouch anal anastomosis (CPAA) groups. Before operation, the patients completed a questionnaire about their bowel habit and chemoradiotherapy condition. This study design was approved at our medical ethics committee in the gastrointestinal research center of the Baqiyatallah (A.S) University of Medical Sciences.

### 3.1. Surgical Technique

The patients were prepared for surgery by gastrointestinal washing using 4Lit polyethylene glycol (PEG) solution 12 hours before surgery. Antibiotic prophylaxis was obtained by oral and IV drugs. After lower abdominal laparotomy, the tumors and mesorectum were completely excised by TA stapler at distal parts, and then according to surgeons’ choice one of the two procedures was done. After resection, the J-pouch was constructed using GIA-60 stapler. We made the side to end coloanal anastomosis by a circular stapler. At the end of procedure, we put a temporary loop ileostomy for all of the patients for the protection of suture line.

### 3.2. Follow-up

All patients were followed up prospectively for evaluation of symptoms and signs, and also for signs of morbidity (e.g. anastomotic leakage) and mortality. After closure of temporary loop ileostomy, functional results were assessed by a questionnaire 6 months postoperatively. In this questionnaire, the daytime, nighttime, and 24-hour stool frequency, the urgency, and bowel incontinence were recorded to assess functional outcome. Continence was classified by the Williams classification (19) Table 1.

**Table 1:** Williams's Classification

Continence Grade	
** Grade I a**	Continent to solids, liquids and flatus
** Grade II b**	Continent to solids and liquids, but not flatus
** Grade III b**	Continent to solid but occasional episodes of liquid incontinence.
** Grade IV c**	Occasional episodes of incontinence of solids and frequent episodes of incontinence of liquids
** Grade V c**	Frequent episodes of incontinence of solids and liquids

^a^Good

^b^Imperfect

^c^Bad

In addition, (QOL) was evaluated by European Organization for Research and Treatment of Cancer (EORTC) QLQ-C30 (20).

### 3.3. Statistical Analysis

All statistical analyses were performed using the SPSS software version 11.5. If a normal data distribution was not accepted by the Kolmogorov-Smirnov test, for comparison of the individual groups of patients, the independent sample t-test or Mann-Whitney U test was applied, respectively. Nominal variables were analyzed using Chi-square test. A two-sided P < 0.05 was considered as significant.

## 4. Results

A total of 88 patients (53 males, 35 females) with rectal cancer underwent LAR with straight coloanal anastomosis (SCAA; n = 47) or colonic J-pouch reconstruction (CPAA; n = 41). In SCAA and CPAA groups, 29 (61.7%) and 24 (58.5%) patients were male, respectively (P = 0.762). There was no significant difference between the mean age of the patients in SCAA and CPAA groups (62.3 ± 13.4, range: 40 - 85 years vs. 63.1 ± 11.9, range: 41 - 82 years; P = 0.795). 10.2% (n = 9) of SCAA group and 13.6% (n = 12) of CPAA group had a history of chemoradiotherapy. The proportion of patients that received preoperative chemotherapy and radiotherapy were comparable in the SCAA and CPAA groups (chemotherapy: 10.6% vs. 9.8%, P = 0.892; radiotherapy: 14.9% vs. 12.2%, P = 0.713). There was one perioperative mortality in the SCAA group (P = 0.348). Anastomotic leakage after SCAA and CPAA were 4.3% vs. 0%, respectively (P = 0.182); which had required a surgical intervention. Details of the functional outcome are shown in Table 2. Although the mean of preoperative stool frequency was the same between the two groups (P = 0.927), but the postoperative defecator function of patients with CPAA was better than patients with SCAA. After operation, the mean bowel movement in 24 hours was significantly higher in SCAA group when compared to CPAA group (5.8 ± 2.2 vs. 3.4 ± 1.4, P < 0.0001). Patients with SCAA had significantly more daytime bowel movements than those in the CPAA group (5.1 ± 1.9 vs. 2.9 ± 1.2, P < 0.0001), but there was no difference between the groups in terms of nighttime bowel movements (0.7 ± 0.9 vs. 0.6 ± 0.8, P = 0.371). A significantly higher incidence of urgency was observed when the SCAA was employed (36.2% vs. 14.6%, OR: 3.3 [1.2 - 9.5], P = 0.022). The mean incontinence scores after CPAA were significantly lower than after SCAA (1.4 ± 0.8 vs. 2.1 ± 1.3, P = 0.007). Also, imperfect and bad continence in patients with SCAA were significantly more common than those in the CPAA group (P = 0.026). Furthermore, there was a significant difference between the SCAA and CPAA groups with regard to overall (QOL) scores (57.2 ± 16 vs. 71 ± 16, P < 0.0001).

**Table 2:** Comparison of Functional Outcome Between the Two Groups

Parameters	SCAA a(n = 47)	CPAA a(n = 41)	P value
**Postoperative bowel movement, Mean ± SD (range**)			
Total 24 hours	5.8 ± 2.2 (3-13)	3.4 ± 1.4 (1-6)	< 0.0001
Daytime	5.1 ± 1.9 (3-12)	2.9 ± 1.2 (1-5)	< 0.0001
Nighttime	0.7 ± 0.9 (0-3)	0.6 ± 0.8 (0-3)	0.37
**Urgency, No. (%) **	17 (36.2)	6 (14.6)	0.022
**Williams incontinence score, Mean ± SD (range)**	2.1 ± 1.3 (1-5)	1.4 ± 0.8 (1-4)	0.007
**Postoperative continence status, No. (%)**			
Good	22 (46.8)	30 (73.2)	
Imperfect	16 (34)	22 (9)	
Bad	9 (19.1)	2 (4.9)	

^a^Abbreviations: CPAA, colonic J-pouch anal anastomosis; SCAA, straight coloanal anastomosis

## 5. Discussion

Our results show that the patients with a colonic J-pouch anal anastomosis (CPAA) had less complications and better functional results and quality of life than patients with a straight coloanal anastomosis (SCAA). The use of a CPAA was shown to reduce stool frequency, urgency, and incontinence, and improve the QOL. Although our follow up are shorter than the other studies but it is really known that frequency of stool passage and continence after pouch becomes better at longer term. This clinical trial explored only one case of perioperative mortality in SCAA group and demonstrated no significant difference between the groups in perioperative mortality. In comparing the rate of perioperative mortality after CPAA and SCAA, Brown (21)obtained an odd ratio of 2.62 [0.56-12.19] in favor of the SCAA, however, like our result, this difference was not significant (P = 0.22). Our data show no significant differences in the rates of anastomotic leaks after SCAA and CPAA. In a randomized clinical trial Hallbook described a significantly lower incidence of anastomotic leakages after CPAA (2%) than after SCAA (15%) (13). Like our study, Sailer (2), Seow (15), Lazorthes (10), and Joo (14) found no significant differences in the incidence of anastomotic complications in prospective randomized studies, and also in their retrospective studies. On average, the reported leakage rate for J-pouch and straight coloanal anastomosis is 9% and 14%, respectively (22). However, our data for SCAA and CPAA are lower than these average leakage rate. It seems that temporary diversion of fecal stream with the routine use of ileostomy can prevent the anastomosis, and may allow the small leaks to heal without symptoms. Functional assessment of our results showed a significant reduction in stool frequency after CPAA compared with SCAA. A number of randomized trials (12-15) are comparing straight versus colonic pouch anastomosis, demonstrating a significant reduction in stool frequency after CPAA when compared with SCAA. Compared with SCAA, reduction in stool frequency after CPAA is attributable to the increase of reservoir capacity and neorectal compliance (23). Recently, an alternative possibility has been presented by Furst, stating that bowel function is improved by reduced bowel motility (24). A higher frequency of stool urgency was found in SCAA than in CPAA, which have been attributed to the loss of rectal reservoir function and a reduced resting anal pressure (25). As in this study, numbers of trials (2, 10, 13) showed that J-pouch anal anastomosis is superior to straight coloanal anastomosis in terms of stool urgency, especially within first postoperative year. This study indicates that CPAA decreases significantly the severity of fecal incontinence. The result of two trials that were done by Hallbook (13) and Seow (15) showed significantly better continence in patients who underwent the CPAA. However, Lazorthes (10) showed no statistically significant difference in fecal incontinence scores between SCAA and CPAA after two years. Similarly, Ho (26) demonstrated no difference between the groups concerning fecal continence. Overall, patients with a CPAA had a better QOL than patients with a SCAA. Although better functional results are not necessarily associated with better QOL, however, it seems that the better bowel function (low frequency, urgency, and incontinence) are associated with higher QOL.esults of this study showed that in patients undergoing LAR for rectal cancers, the CPAA is superior to SCAA in terms of stool frequency, urgency, and incontinence. Overall, postoperative complications rates are similar between both techniques. Therefore, the CPAA can be a better choice for reconstruction after a LAR.
